# Loss of protohaem IX farnesyltransferase in mature dentate granule cells impairs short‐term facilitation at mossy fibre to CA3 pyramidal cell synapses

**DOI:** 10.1113/JP273581

**Published:** 2017-03-15

**Authors:** Sam A. Booker, Graham R. Campbell, Karolina S. Mysiak, Peter J. Brophy, Peter C. Kind, Don J. Mahad, David J. A. Wyllie

**Affiliations:** ^1^ Centre for Integrative PhysiologyUniversity of EdinburghHugh Robson Building, George SquareEdinburghEH8 9XDUK; ^2^ Patrick Wild CentreUniversity of EdinburghHugh Robson Building, George SquareEdinburghEH8 9XDUK; ^3^ Centre for Clinical Brain SciencesUniversity of EdinburghChancellor's Building, 49 Little France CrescentEdinburghEH16 4SBUK; ^4^ Centre for NeuroregenerationUniversity of EdinburghChancellor's Building, 49 Little France CrescentEdinburghEH16 4SBUK; ^5^ Centre for Brain Development and RepairInstitute for Stem Cell Biology and Regenerative MedicineBangalore560065India

## Abstract

**Key points:**

- Neurodegenerative disorders can exhibit dysfunctional mitochondrial respiratory chain complex IV activity.
- Conditional deletion of cytochrome *c* oxidase, the terminal enzyme in the respiratory electron transport chain of mitochondria, from hippocampal dentate granule cells in mice does not affect low‐frequency dentate to CA3 glutamatergic synaptic transmission.
- High‐frequency dentate to CA3 glutamatergic synaptic transmission and feedforward inhibition are significantly attenuated in cytochrome *c* oxidase‐deficient mice.
- Intact presynaptic mitochondrial function is critical for the short‐term dynamics of mossy fibre to CA3 synaptic function.

**Abstract:**

Neurodegenerative disorders are characterized by peripheral and central symptoms including cognitive impairments which have been associated with reduced mitochondrial function, in particular mitochondrial respiratory chain complex IV or cytochrome *c* oxidase activity. In the present study we conditionally removed a key component of complex IV, protohaem IX farnesyltransferase encoded by the *COX10* gene, in granule cells of the adult dentate gyrus. Utilizing whole‐cell patch‐clamp recordings from morphologically identified CA3 pyramidal cells from control and complex IV‐deficient mice, we found that reduced mitochondrial function did not result in overt deficits in basal glutamatergic synaptic transmission at the mossy‐fibre synapse because the amplitude, input–output relationship and 50 ms paired‐pulse facilitation were unchanged following *COX10* removal from dentate granule cells. However, trains of stimuli given at high frequency (> 20 Hz) resulted in dramatic reductions in short‐term facilitation and, at the highest frequencies (> 50 Hz), also reduced paired‐pulse facilitation, suggesting a requirement for adequate mitochondrial function to maintain glutamate release during physiologically relevant activity patterns. Interestingly, local inhibition was reduced, suggesting the effect observed was not restricted to synapses with CA3 pyramidal cells via large mossy‐fibre boutons, but rather to all synapses formed by dentate granule cells. Therefore, presynaptic mitochondrial function is critical for the short‐term dynamics of synapse function, which may contribute to the cognitive deficits observed in pathological mitochondrial dysfunction.

## Key points

- Neurodegenerative disorders can exhibit dysfunctional mitochondrial respiratory chain complex IV activity.
- Conditional deletion of cytochrome *c* oxidase, the terminal enzyme in the respiratory electron transport chain of mitochondria, from hippocampal dentate granule cells in mice does not affect low‐frequency dentate to CA3 glutamatergic synaptic transmission.
- High‐frequency dentate to CA3 glutamatergic synaptic transmission and feedforward inhibition are significantly attenuated in cytochrome *c* oxidase‐deficient mice.
- Intact presynaptic mitochondrial function is critical for the short‐term dynamics of mossy fibre to CA3 synaptic function.

**Abbreviations**
AD: Alzheimer's disease
COX: cytochrome *c* oxidase
DGC: dentate granule cell
EPSC: excitatory postsynaptic current
GCL: granule cell layer
IN: interneuron
IPSC: inhibitory postsynaptic current
MF: mossy fibre
MFB: mossy fibre bouton
MS: multiple sclerosis
PC: pyramidal cell
PPR: paired‐pulse ratio
STF: short‐term facilitation

## Introduction

Neurodegenerative disorders are highly debilitating and progressive neurological diseases, typified by symptoms localized to the peripheral and central nervous system (CNS) (Simon & Johns, 1999). Mitochondrial defects are implicated in normal ageing and the pathogenesis of a number of neurodegenerative disorders where the hippocampus harbours cells deficient in respiratory chain complexes or enzymes, particularly the granule cells of the dentate gyrus (DG) (Nagy *et al*. 1999; Martin *et al*. 2016) and the molecular layer of the DG (Simonian & Hyman, 1993). A causal link has been suggested between the mitochondrial energy transfer system and diseases such as Alzheimer's disease (AD), Parkinson's disease (PD), multiple sclerosis (MS), epilepsy and primary mitochondrial disorders such as Leigh syndrome with loss of mitochondrial function linked to reduced neuronal and glial function (reviewed in Beal, 1995; Lin & Beal, 2006; Diaz, 2010; Waldbaum & Patel, 2010; Campbell *et al*. 2014). The terminal component of the electron transport chain is cytochrome *c* oxidase (COX or mitochondrial respiratory chain complex IV), of which a major component is protohaem IX farnesyltransferase, encoded by the *COX10* gene. Previous research has shown that conditional loss of *COX10* in oligodendrocytes leads to glycolytic metabolism of ATP leading to increased lactic acid build up in the cerebral cortex (Funfschilling *et al*. 2012), with a phenotype akin to MS (Campbell *et al*. 2014). However, a few studies have shown that loss of mitochondrial function leads to profound effects on synaptic transmission in the CNS. Lee *et al*. (2012) showed that aberrant mitochondrial function in granule cells of the DG in a mouse model of AD leads to short‐term plasticity deficits in the presynaptic axon, which is consistent with earlier studies (Tang & Zucker, 1997). Furthermore, it has been shown that the presence of mitochondria is a prerequisite for the development and plasticity of postsynaptic dendritic spines (Li *et al*. 2004).

The glutamatergic dentate granule cell (DGC) synapse onto CA3 pyramidal cells (CA3 PCs) in the hippocampus is one of the most widely studied of the central synapses. This very large (3–5 μm) mossy‐fibre bouton (MFB) synapse serves as the main transfer of synaptic information from the DG to the CA3, containing multiple mitochondria due to their large size and high energy requirement (Hamlyn, 1962). MFBs in stratum (str.) lucidum of CA3 form multiple postsynaptic contacts with CA3 PC apical dendrites and have distinct synaptic features, notably short‐term plasticity, which is believed to act as a ‘detonator’ for the firing of CA3 PCs, following short bursts of high‐frequency firing of DGCs. Interestingly, at least 10 times more synapses are formed by DGCs onto local GABAergic interneurons in the hilus and CA3 (Acsády *et al*. 1998), indicative of tight control of both glutamatergic and GABAergic neurotransmission from DGCs. Despite much being known regarding the short‐term dynamics of MFB glutamate release, little is known regarding the function of this synapse when mitochondria are disrupted in DGCs, as previous studies have only focused on presynaptic potentiation following prolonged high‐frequency stimuli.

To address whether loss of *COX10*, and thus mitochondrial respiratory chain complex IV activity, leads to compromised synaptic transmission and short‐term plasticity in the MF pathway we performed whole‐cell electrophysiological recordings from identified CA3 PCs and DGCs in acute hippocampal slices from a mouse model, where an inducible *COX10* deletion was performed preferentially from neurons using a *Thy‐1* promoter, preferentially expressed in the DG. Recordings of evoked synaptic currents showed that there is a requirement for intact mitochondrial function in the maintenance of short‐term plasticity at MFBs, and the recruitment of feedforward inhibition, onto CA3 PCs.

## Methods

### Ethical approval

Experiments conducted during the course of this study received approval from the University of Edinburgh's Local Ethical Review Board. Animal breeding and maintenance and experimental procedures were performed in accordance with the UK Animals (Scientific Procedures) Act 1986 under the authority of Project Licences 70/7872 (D.J.M.) and 60/4290 (D.J.A.W.).

### Animal generation

C57/Bl6J double transgenic mice were used in the current study, expressing *COX10^flox/flox^* (Funfschilling *et al*. 2012) and an inducible Cre‐recombinase under the *Thy‐1* promotor (*Thy1*‐cre/ERT2) (Zonta *et al*. 2011). Mice were housed under a 12 h light–dark cycle and given access to food and water *ad libitum*. Deletion of the *COX10* gene was induced with tamoxifen (10 mg ml^–1^ dissolved in sunflower oil and administered i.p.) at postnatal week 5–7, after which time mice developed the mitochondrial biochemical deficiency at 11 postnatal weeks and became symptomatic at 17 postnatal weeks. In a subset of experiments *Thy1*‐cre/ERT2 selectivity was confirmed by crossing these mice with a yellow fluorescent protein (YFP) reporter line (*n = *4 *Thy1*‐cre mice, *n = *3 *Thy1*‐cre lacking mice). A total of 16 (8 control, 8 mutant) mice at 15 postnatal weeks were used in the present study. All experiments were performed in a pairwise fashion on a single experimental day, where possible.

### Acute hippocampal slice preparation

Following tamoxifen induction and onset of symptoms, hippocampal slices were prepared as described previously (Booker *et al*. 2014). Briefly, following cervical dislocation brains were rapidly removed and placed in carbogenated (95% O_2_–5% CO_2_), ice‐cold sucrose–artificial cerebrospinal fluid (ACSF) (in mm: 87 NaCl, 2.5 KCl, 25 NaHCO_3_, 1.25 NaH_2_PO_4_, 25 glucose, 75 sucrose, 7 MgCl_2_, 0.5 CaCl_2_). Transverse hippocampal slices (300 μm thick) from the ventral third of the hippocampus were sliced on a vibrating blade microtome (VT1200S, Leica Instruments, Germany), then transferred to a submerged holding chamber containing carbogenated sucrose–ACSF. Slices were incubated at 35°C for 30 min and then stored at room temperature until required.

### Whole‐cell patch‐clamp recordings

Slices were transferred to a submerged recording chamber and superfused with carbogenated (95% O_2_/5% CO_2_), normal ACSF (in mm: 125 NaCl, 2.5 KCl, 25 NaHCO_3_, 1.25 NaH_2_PO_4_, 25 glucose, 1 MgCl_2_, 2 CaCl_2_, 1 sodium pyruvate, 1 ascorbic acid) at 32–34°C. The hippocampus was visualized with an upright microscope (BX51‐WI, Olympus, Hamburg, Germany), working under infrared differential interference contrast (IR‐DIC) optics with an IR‐CCD camera (Orca2, Hamamatsu, Japan; HCI software, Hamamatsu, Japan). Whole‐cell recording electrodes were produced from filamented borosilicate glass capillaries (1.5 mm outer, 0.7 mm inner diameter) on a horizontal puller (P‐97, Sutter Instrument Co., Novato, CA, USA). Pipettes were filled with a potassium gluconate‐based solution (in mm: 142 potassium gluconate, 4 KCl, 2 MgCl_2_, 0.1 EGTA, 10 Hepes, 2 Na_2_‐ATP, 0.3 Na_2_‐GTP, 1 Na_2_‐phosphocreatinine, 0.1% biotinylated‐lysine (Biocytin, Invitrogen, UK); pH 7.3, 290–310 mosmol l^–1^), with a final resistance of 2–5 MΩ.

Recordings were performed using a Multiclamp 700B amplifier (Molecular Devices, Sunnyvale, CA, USA). Voltage and current signals were low‐pass filtered at 10 and 2 kHz respectively, using the built in Bessel filter of the amplifier, digitised at 20 kHz (Digidata 1440, Molecular Devices) and acquired with pCLAMP software (Molecular Devices). Data were analysed offline using the Stimfit software package (courtesy of C. Schmidt‐Hieber; http://www.stimfit.org; Guzman et al. 2014).

Whole‐cell patch‐clamp recordings were achieved and the intrinsic physiology of neurons was recorded in current‐clamp mode with a family of current pulses (–500 to 500 pA, 100 pA steps, 500 ms duration) and a series of small hyperpolarizing steps (–10 pA, 500 ms duration, 30 repetitions). All synaptic currents were recorded in voltage‐clamp, at a holding potential of ‒65 mV. To ascertain the properties of spontaneous excitatory activity, a 1–2 min continuous recording was made. Evoked excitatory postsynaptic currents (EPSCs) were elicited via extracellular stimulation with a bipolar nichrome electrode (100 μm diameter) placed in str. lucidum of CA3, 50–150 μm rostral from the recorded CA3 PC (duration 0.1 ms). An initial input–output relationship was performed across a stimulus range of 0–25 V, in 5 V increments and three responses were collected at each stimulus level. In a subset of experiments, minimal stimulation was performed at the lowest stimulation intensity that produced a synaptic response with apparent failures of transmission. Synaptic stimulation experiments were performed at near maximal response amplitude with a mean intensity of 19.8 ± 1.8 V, applied from a constant‐voltage isolated stimulus generator (Digitimer DSA2, Cambridge, UK). Synaptic properties were analysed by an applied paired‐pulse protocol (2 stimuli, 50 ms interval), and at least 20 traces collected. Short‐term plasticity was interrogated with trains of 10 stimuli delivered at 5, 10, 20, 50 and 100 Hz (20 s inter‐sweep interval); at least 10 traces were collected for each frequency tested. To confirm that the synaptic response originated from the MFB, (2*S*,2′*R*,3′*R*)‐2‐(2′,3′‐dicarboxycyclopropyl)glycine (DCG‐IV; 5 μm) was applied to the bath for 5 min, while recording paired‐pulse responses. Cells were excluded if DCG‐IV failed to inhibit the synaptic response by at least 40%. Series resistance (*R*_S_) was constantly monitored throughout the recordings, but was not compensated. Recordings were abandoned if initial *R*_S_ exceeded 30 MΩ or changed by more than 20% over the time course of the recording.

In a subset of recordings a caesium gluconate‐based internal solution (140 caesium gluconate, 4 CsCl, 0.1 EGTA, 10 Hepes, 2 Mg‐ATP, 0.3 Na_2_‐GTP, 1 Na_2_‐phosphocreatinine, 5 QX‐314; pH 7.35, 305 mosmol l^–1^) was used to record the GABA:AMPA ratio of MF inputs. Synaptic stimulation was performed as above with the same stimulation protocols employed. Voltage clamp recordings were made at –70 and at 0 mV to dissect AMPA and GABA_A_ currents, respectively. Following recording at 0 mV, 10 μm CNQX and 50 μm APV were applied to the bath, to block AMPA and NMDA receptor‐mediated transmission and reveal the monosynaptic GABA_A_‐mediated IPSC. Finally, the GABA_A_ receptor blocker picrotoxin (50 μm) was applied to the bath, to confirm receptor selectivity. Feedforward inhibition was estimated following subtraction of the CNQX/APV insensitive IPSC from the total IPSC at 0 mV; this was then normalized to the AMPA current to give the GABA:AMPA ratio. To determine whether the total inhibitory envelope was altered, we also measured the charge transfer, as the integral of the EPSC/IPSCs, which was normalized as a GABA:AMPA ratio as well. Liquid junction potentials were not compensated; however, in all experiments reversal of EPSCs was tested and observed to be 0 mV.

Spontaneous EPSCs were analysed based upon the fitting of the responses to a triexponential curve‐fit, which was then template matched (Clements & Bekkers, 1997) to 60 s of continuous recording. Detected EPSCs were excluded if their amplitude was less than 2 standard deviations of the baseline noise. Peak EPSC amplitude of both synaptic and spontaneous events was measured over a 1 ms time window from the 5 ms baseline directly preceding the stimulus. Short‐term plasticity was measured as compared to the first synaptic response elicited by a given protocol. All physiological properties were analysed using the Stimfit software package (Guzman *et al*. 2014).

### Morphological characterization

Following recording, slices were immediately immersion‐fixed in 4% paraformaldehyde in phosphate buffered saline (PBS; 0.1 PB + 0.9% NaCl), overnight at 4°C. Slices were then rinsed copiously in PBS and incubated overnight at 4°C in a solution containing streptavidin conjugated to Alexafluor 633 nm (1:500), Triton X‐100 (0.3%) and NaN_3_ (0.05%). Slices were then rinsed liberally in PBS, then mounted on glass slides with a polymerizing anti‐fade mounting medium (Vectashield, Vector Laboratories) and cover‐slipped. Neurons were imaged on a scanning confocal microscope (Axioskop, Zeiss, Germany) and *z*‐stacks (1 μm step size, 1024 pixel resolution) taken over the entire somatodendritic axis of the neuron. Neurons were three‐dimensionally reconstructed offline using the Simple Neurite Tracer plugin of FIJI (Longair *et al*. 2011) and then Sholl analysis performed on the segmented skeleton.

### Histochemical and immunohistochemical detection of mitochondrial respiratory chain complexes

COX and succinate dehydrogenase (SDH) activity were determined as follows: COX media (100 μm cytochrome *c*, 4 mm diaminobenzidine tetrahydrochloride and 20 μg ml^–1^ catalase in 0.1 m phosphate buffer at pH 7.0) was applied to snap‐frozen brain tissue, washed in PBS before SDH medium (130 mm sodium succinate, 200 mm phenazine methosulphate, 1 mm sodium azide and 1.5 mm nitroblue tetrazolium in 0.1 m phosphate buffer pH 7.0) was applied for a further 20 min at 37°C as previously described (Johnson *et al*. 1993, Campbell *et al*. 2011). Furthermore, a main catalytic subunit of complex IV (COXI) as well as complex II (C‐II 70 kDa) were detected together with a neuronal marker using standard immunofluorescence techniques, as previously described (Campbell *et al*. 2011). The threshold for identifying DG neurons with COXI was determined by the mean plus 2 standard deviations of the mutant DG neurons that qualitatively lacked COXI. Any cell that fell 2 standard deviations below this mean was therefore considered to be COXI deficient.

### RNA probe generation and *in situ* hybridization

cDNA was generated by reverse transcription polymerase chain reaction (RT‐PCR) using RNA from mouse brain. Primers were designed to span exon 6 of the *COX10* gene generating a 531 base pair product. Forward primer: 5′‐GTGCCGTTCGACTCAAACAT‐3′; reverse primer: 5′‐GATGGGGAGGGAGATGACAG‐3′. The RT‐PCR product was cloned into plasmid pSC‐A‐amp‐kan and sequenced to confirm insertion of PCR product and orientation. Digoxigenin‐labelled RNA (DIG‐RNA) probes were transcribed in the anti‐sense orientation using T7 RNA polymerase. *In situ* hybridization was carried out on fresh frozen cryostat sections of mutant (*n = *3) and WT mice (*n = *3). Sections were briefly fixed in 4% paraformaldehyde and a hybridization buffer (50% deionized formamide, 1× SSC (300 mm NaCl, 30 mm citric acid trisodium salt), 5 mm EDTA pH 8, 0.1% Tween 20, Denhardt's solution, 2.76% dextran sulfate, 2 mg heparin, 0.1% Chaps, 2 mg tRNA in H_2_O) was applied for 1 h at 70°C before the DIG‐RNA probe was applied at 1:1000 in hybridization buffer overnight at 70°C. Sections were then washed in 1× SSC and MABT (100 mm maleic acid, 150 mm NaCl, 0.5% Tween 20) and kept in a blocking solution of 2% normal sheep serum for 3 h at room temperature. Anti‐DIG antibody was applied overnight at 4°C at a concentration of 1:2000. Sections were washed in MABT and NTMT (100 mm NaCl, 100 mm Tris, 200 mm MgCl_2_ and 0.1% Tween) and were left in a staining solution of NBT and BCIP (Sigma) overnight at room temperature, washed in PBS briefly and mounted in glycerol.

### Statistical analysis

All data are shown as the mean ± SEM. Data were averaged for each animal used, thereby removing pseudo‐replication, and thus oversampling, of the data. Average data throughout are presented as the average of each animal or group of animals. Statistical tests were performed on unpaired data using either the Mann–Whitney non‐parametric test, or two‐way ANOVA. For comparison of cumulative probability, an *F*‐test was employed. Data were deemed statistically significant if *P < *0.05. Data were analysed using Prism (GraphPad Software, La Jolla, CA, USA).

## Results

### Loss of Complex IV in DGCs minimally alters CA3 PC morphology and intrinsic excitability

**Figure 1:**
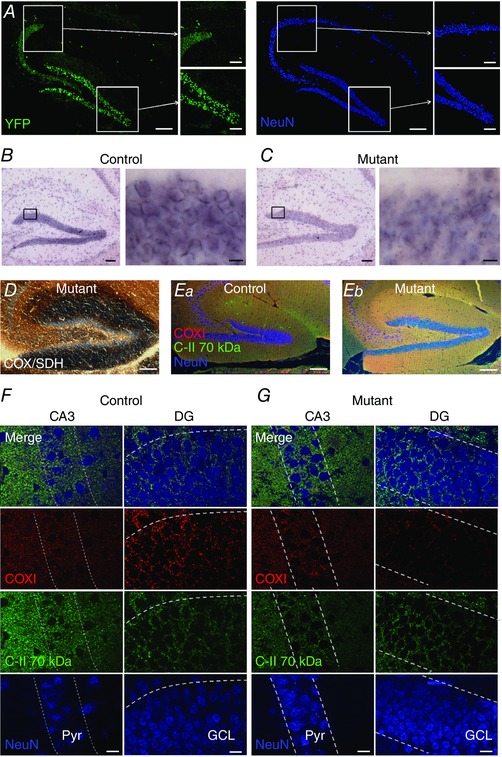
Complex IV‐deficient neurons are specifically localized to the DG *A*, overview of Thy1‐cre expression in the hippocampus, resulting from crossing with a YFP reporter line (green); neurons are shown for reference through NeuN labelling (blue pseudocolour). Scale bar: 100 μm (main images) and 50 μm (insets) *B*, low (left) and high (right) magnification light micrographs of the DG from control mice labelled with *in situ* hybridization to COX10 RNA. Scale bar: 100 μm (left) and 10 μm (right). *C*, the same as *B*, except in the *COX10*‐deficient (mutant) mice, and with reduced RNA aggregations at the soma of DGCs. *D*, sequential complex IV (COX) and complex II (SDH) histochemistry on snap‐frozen hippocampus from mutants shows complex IV‐deficient cells lacking enzyme activity (stained blue) in the DG. Scale bar: 100 μm. *E*, low power image of immunofluorescence labelling against complex II 70 KDa subunit (C‐II 70 kDa, green) and the subunit I of complex IV (COXI, red) with NeuN (blue) hippocampal sections from control and *COX10*‐deficient mice. Scale bar: 100 μm. Note the absence of labelling from the GCL of DG and str. lucidum of CA3. *F*, high power images of the CA3 str. pyramidale (Pyr) and GCL in DG from control mice with intact COXI (red), but with the C‐II subunit present (green), overlaid with NeuN for comparison (blue). Cell body layers are delineated by dashed lines for reference; scale bar: 10 μm. *G*, DGCs from mutant mice lack COXI, according to the same scheme as *E*.

To confirm that our *Thy1*‐cre line showed specific DG expression, we crossed a *Thy1*‐cre mouse line with a YFP reporter line, which indicated selective expression of *Thy1*‐cre in the dentate region of the hippocampus (Fig. 1 *A*). The *Thy1*‐cre that we utilized has been previously shown to be selectively expressed in specific neuronal populations (Zonta *et al*. 2011). The current data suggest that it was preferentially expressed by DGCs in the hippocampus. *In situ* hybridization for COX10 RNA indicates loss of positive staining in the majority of DGCs when compared to WT (Fig. 1 *B* and *C*). In accord with our *in situ* hybridization data, COX/SDH histochemistry revealed that 70.2 ± 5.0% of DGCs lacked COX activity (*n = *4) compared to only 2.0 ± 0.4% of CA3 PCs in mutant mice (Fig. 1 *D*, *P < *0.0001, Student's *t* test). No loss of COX activity was apparent in *Thy1*‐cre WT mice in either DGCs or CA3 PCs (*n = *3 mice). Using immunofluorescence labelling in the tamoxifen‐induced mutant mice, we observed selective loss of COXI from DGCs (Fig. 1 *E*–*G*); 78.0 ± 3.3% of DGCs showed loss of COXI (*n = *4 mice), whereas in the same mice, only 2.2 ± 0.2% of CA3 PCs showed loss of COXI (*P < *0.0001, Student's *t* test). No loss of COXI was observed in *Thy1*‐cre WT mice in either DGCs or CA3 PCs (*n = *3 mice). Complex II (C‐II 70 kDa) remained intact in all DGCs and CA3 PCs (Fig. 1 *E–G*). Our data confirm that the specificity of the complex IV deletion was to DGCs, with minimal loss of expression in the CA3 region.

**Figure 2:**
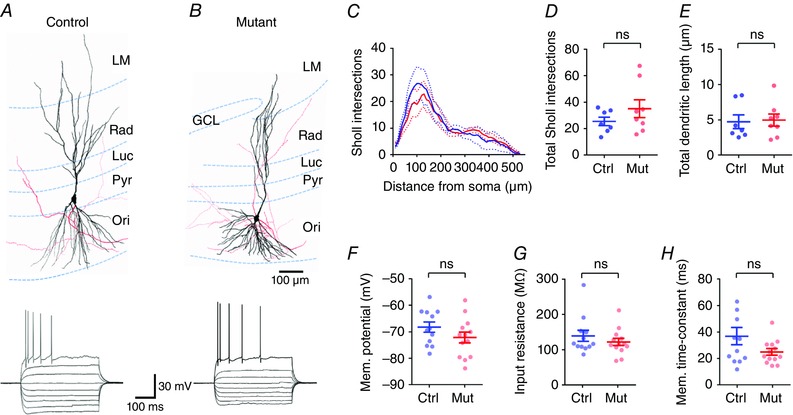
Morphology and intrinsic excitability are mostly normal in CA3 PCs when *COX10* is removed from DGCs *A* and *B*, upper, 3‐dimensional reconstructions of CA3 PCs from control (left) and *COX10*‐deficient mice (mutant; right), showing the somatodendritic axis (black) and the axonal arborization (red). Lower, representative voltage responses of CA3 PCs, following –250 pA to +250 pA current injections (50 pA steps, 500 ms duration). *C*, Sholl analysis of CA3 PCs from control (blue, *n = *4 cells) and *COX10*‐deficient mice (red, *n = *6 cells). Note that CA3 PCs from mice lacking *COX10* in DGCs showed no difference from control cells. *D* and *E*, concomitantly there was no difference in either the number of total Sholl intersections (*D*), or the total dendritic length (*E*). *F*–*H*, there was no observable difference in resting membrane potential (*F*), input resistance (*G*), or membrane time constant (*H*) between control mice and *COX10*‐deficient mice, suggesting normal intrinsic physiology. Statistics shown: ns, *P > *0.05, Student's 2‐tailed *t* test.

To determine if loss of complex IV from DGCs resulted in increased excitability in the downstream CA3 microcircuit, as expected from studies into the effect of mitochondrial dysfunction on epileptiform activity (reviewed in Patel, 2004), we performed whole‐cell recordings from identified CA3 PCs and recorded intrinsic physiology and performed morphological analysis of CA3 PCs. Recorded neurons were filled with biocytin (0.1% w/v), included in the intracellular solution, and identified following recording using streptavidin conjugated to a fluorophore. All neurons recorded in the current study were identified as CA3 PCs (Fig. 2 *A* and *B*). CA3 PCs had somata located in str. pyramidale or in proximal str. oriens, with a single large calibre apical dendrite passing into str. radiatum, which bifurcated and formed a tuft in str. lacunosum‐moleculare (LM), and basal dendrites in str. oriens. The apical dendrite in str. lucidum possessed many large thorny excrescences, indicative of MFBs. The axon of CA3 PCs typically arose from the soma or a proximal dendrite and formed a sparse local arbor, with several collaterals passing into str. radiatum forming the Schaffer collaterals. A subset of CA3 PCs were reconstructed from both control (*n = *4) and complex IV‐deficient (*n = *6) mice; however, neither the dendritic complexity (Fig. 2 *C* and *D*), as measured from Sholl analysis, nor the total dendritic length (Fig. 2 *E*) was different between the two groups of cells.

**Figure 3:**
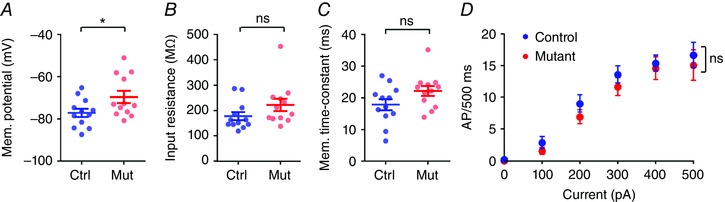
DGCs have depolarized membranes, but normal passive and active properties in *COX10*‐deficient mice *A*, the resting membrane potential of DGCs was depolarized in *COX10*‐deficient mice, compared to control mice. *B* and *C*, despite the changed membrane potential, there was no observable difference in input resistance (*B*) or membrane time constant (*C*). *D*, from a set holding potential of –70 mV, there was no difference in the ability of DGCs in *COX10*‐deficient mice to fire trains of action potentials in response to depolarizing current steps. Statistics shown: ns, *P > *0.05; ^*^ *P < *0.05.

As neurons may undergo homeostatic changes in intrinsic excitability to compensate for hyperexcitability (O'Leary *et al*. 2010), we next asked whether there were overt changes in the intrinsic physiology of CA3 PCs in *COX10*‐deficient DGCs. The intrinsic physiology of CA3 PCs was normal in complex IV‐deficient mice as resting membrane potential (Fig. 2 *F*), input resistance (Fig. 2 *G*), membrane time constant (Fig. 2 *H*), and maximal action potential discharge were all similar to that of control mice (see Table 1). Consistent with this, action potential properties were similar between both groups of mice. As Thy1‐cre is selectively expressed in DGCs as opposed to CA3 PCs (Fig. 1), we next asked whether DGCs themselves showed altered excitability. Indeed, loss of *COX10* leads to a strongly depolarized resting membrane potential (Fig. 3 *A*), which was –77.1 ± 2.0 mV in control mice, but –69.6 ± 2.8 mV in *COX10*‐deficient mice (*n = *12 cells from 5 mice per group). Interestingly there was no change in input resistance (Fig. 3 *B*), membrane time constant (Fig. 3 *C*), or the ability of DGCs to fire action potentials (Fig. 3 *D*; as measured from –70 mV).

**Table 1:** Summary of intrinsic physiological properties of CA3 PCs in control and *COX10*‐deficient mutant mice

	Control (12 mice)	Mutant (13 mice)	*P*
Membrane potential (mV)	−68.3 ± 1.9	−72.2 ± 2.06	0.18
Input resistance (MΩ)	138.6 ± 15.4	121.5 ± 10.3	0.36
Membrane time constant (ms)	37.0 ± 6.5	25.1 ± 2.5	0.09
Rheobase (pA)	320 ± 54	400 ± 40	0.22
Action potential threshold (mV)	−41.9 ± 1.6	−40.2 ± 2.2	0.43
Maximum discharge frequency (Hz)	13.0 ± 1.9	13.6 ± 1.7	0.80

Data are shown as mean ± SEM. Statistics are shown from Mann–Whitney non‐parametric tests.

### Loss of complex IV in DGCs increases excitability in CA3

**Figure 4:**
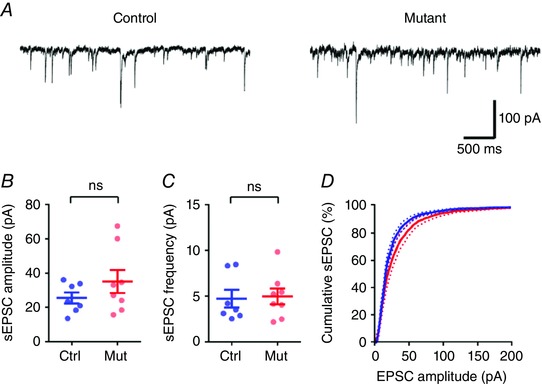
Spontaneous excitability is increased at the *COX10*‐depleted DG–CA3 synapse *A*, representative spontaneous EPSC (sEPSC) recordings from CA3 PCs from control (left) and *COX10*‐deficient mice (right). *B* and *C*, neither sEPSC average amplitude (*B*) nor frequency (*C*) was substantially increased in *COX10*‐deficient (*n = *8 mice, blue) or control mice (*n = *7 mice, red). *D*, the cumulative distribution of sEPSC amplitude, however, displayed a clear tendency towards larger EPSCs in the *COX10*‐deficient mice. Statistics shown: ns, *P > *0.05; ^***^ *P < *0.001.

To determine if the CA3 microcircuit showed increased excitability, we next measured spontaneous excitatory postsynaptic currents (sEPSCs) recorded in CA3 PCs. All synaptic currents were recorded from a holding potential of ‒65 mV and resulted from activation of ionotropic glutamate receptors, not GABA_A_ receptors, due the reversal potential for Cl^−^ of ‒75 mV (calculated and measured, not shown), which resulted in large outward currents which were excluded from this analysis. In control mice (*n = *11 cells from 7 mice), CA3 PCs possessed a high frequency of sEPSCs (4.7 ± 1.0 Hz) with a large average amplitude (25.5 ± 3.3 pA; Fig. 4). Compared to control mice, mice lacking complex IV in DGCs (*n = *13 cells from 8 mice) displayed no apparent difference in either the amplitude (35.1 ± 6.7 pA; *P = *0.39, Fig. 4 *B*) or the frequency (5.0 ± 0.9 Hz; *P = *0.83, Fig. 4 *C*) of sEPSCs in CA3 PCs. Interestingly, the cumulative probability of sEPSC amplitude showed a tendency towards larger sEPSCs in the mutant mice as evidenced by a clear difference in the resulting probability distributions (Fig. 4 *D*; *P < *0.0001, Kolmogorov–Smirnov test). These data suggest that although basal activity within CA3 is unchanged, there is a tendency towards larger synaptic responses, which is not reflected in the mean of all cells. However, as spontaneous EPSCs arising from the low‐release probability MFB are likely to form a minority of the spontaneous activity, it is possible that the observed difference in cumulative EPSC amplitude may have an alternative source.

### Basic MF transmission is unaffected by the lack of complex IV in DGCs

**Figure 5:**
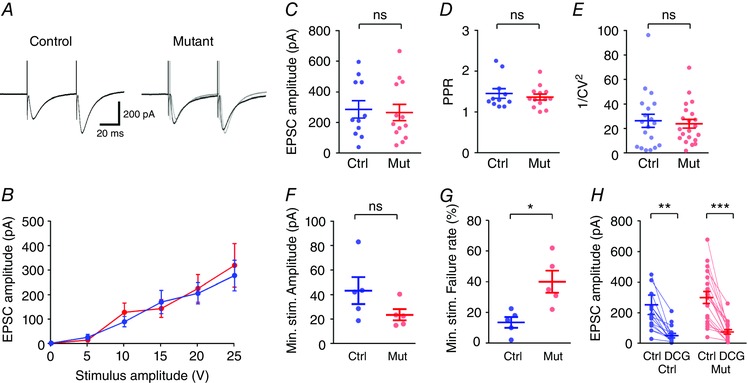
Basal synaptic transmission at the MFB is normal in the *COX10*‐deficient mouse *A*, representative traces showing EPSCs elicited in response to str. lucidum stimulation under paired‐pulse protocols for control (left) and *COX10*‐deficient mice (right); control traces are shown in grey for comparison. *B*, there was no difference in the input–output relationship between control (blue) and mutant mice (red). *C*–*E*, EPSC amplitude (*C*), paired‐pulse ration (*D*) and 1/CV^2^ (*E*) all showed no difference between control and mutant mice. *F*, minimal stimulation of str. lucidum resulted in unitary EPSCs in CA3 PCs, of which there was no observable difference in amplitude between genotypes. *G*, the failure rate of unitary connections was quantified for control and *COX10*‐deficient mice. *H*, evoked EPSCs in all cells recorded were strongly inhibited by bath application of DCG‐IV (5 μm). Statistics shown: ns, *P > *0.05; ^*^ *P < *0.05; ^**^ *P < *0.01; ^***^ *P < *0.001.

As sEPSCs arise from a mixed population of synapses, of which MFBs make up a minority, we next attempted to ascertain whether EPSCs arising from direct stimulation of the MF tract were altered by the loss of complex IV. Whole‐cell patch clamp recordings from CA3 PCs were combined with extracellular stimulation of the proximal (< 150 μm) str. lucidum, Electrical stimulation delivered under a paired‐pulse protocol (50 ms interval) resulted in robust synaptic responses in CA3 PCs, which when recorded in voltage clamp (Fig. 5 *A*) showed an increase in EPSC amplitude in response to increased stimulation intensity (Fig. 5 *B*). All experiments presented are from 13 *COX10*‐deficient mice (21 cells) and 11 control mice (17 cells); one to two cells were collected per mouse. In control mice, the evoked EPSC had an average amplitude of 289.2 ± 60.0 pA, with a paired‐pulse ratio (PPR) of 1.46 ± 0.12, consistent with the strong facilitation previously observed at the MFB (Fig. 5 *C* and *D*). Mice deficient in *COX10* showed a similar EPSC amplitude of 268.4 ± 53.0 pA (*P = *0.79) and PPR of 1.37 ± 0.07 (*P = *0.52; Fig. 5 *C* and *D*). Likewise the overall variability of the EPSC amplitude, as measured as 1/CV^2^, was similar between control (26.4 ± 5.4) and *COX10*‐deficient mice (24.0 ± 3.6, *P = *0.70; Fig. 5 *E*). In a subset of experiments (*n = *8 cells from 5 mice, per group), we performed minimal stimulation of mossy fibres, to produce an all‐or‐nothing unitary synaptic response. The average amplitude of the minimal stimulation response (from non‐failures) was not different between control (43.1 ± 10.9 pA) and *COX10*‐deficient (23.6 ± 4.5 pA) mice (*P = *0.14, *t* test; Fig. 5 *F*), which had similar 1/CV^2^ values (control: 2.3 ± 0.3, mutant: 2.7 ± 0.6, *P = *0.49). Surprisingly, the failure rate of minimally stimulated EPSCs was substantially different between control (13.5 ± 3.5%) and *COX10*‐deficient mice (40.0 ± 7.1%; *P = *0.01, *t* test; Fig. 5 *G*), indicating a possible reduced release probability at the direct MFB synapse onto CA3 PCs.

To confirm that EPSCs in both groups were produced by direct stimulation of the MFs, we applied the selective Group 2/3 mGluR agonist DCG‐IV (5 μm) to the perfusing ACSF, which reduced the EPSC amplitude by 74.1 ± 5.1% and 74.0 ± 3.9% in control and complex IV‐deficient mice, respectively (*P = *0.56, Fig. 5 *H*). From these data, basal synaptic transmission at the MFB was broadly similar in amplitude in both control and *COX10*‐deficient mice, but release probability appeared to be reduced.

### Short‐term facilitation at the MF synapse is impaired at high frequencies

**Figure 6:**
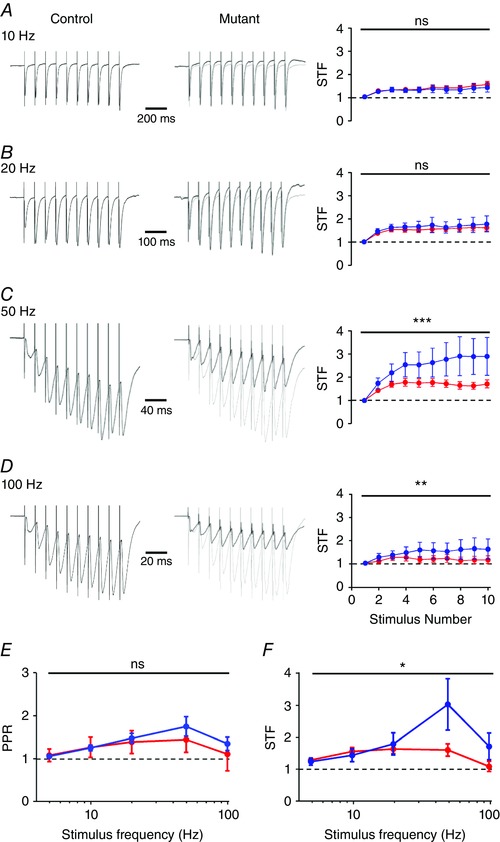
*COX10*‐deficient MF terminals show deficits in short‐term plasticity at high frequencies *A*–*D*, representative trains of 10 stimuli delivered at 10–100 Hz (indicated) to str. lucidum from control (left) and *COX10*‐deficient (middle) mice; note traces are scaled to the peak amplitude of the first EPSC. Right, the degree of short‐term facilitation (STF) as measured as EPSC amplitude compared to the first EPSC. Data are shown for control (blue) and *COX10*‐deficient mice (red); note that EPSCs recorded in CA3 PCs do not show sustained facilitation at high frequencies. *E*, quantification of paired pulse ratio (PPR; second to first EPSC) over the range of frequencies tested. *F*, STF ratio as measured as last to first EPSC as measured over the same frequency range. Note the strong depression of STF from 20 to 100 Hz. Statistics shown: ns, *P > *0.05; ^*^ *P < *0.05; ^**^ *P < *0.01; ^***^ *P < *0.001.

As complex IV is intrinsically involved in the energy balance of neurons, we next asked whether the MF–CA3 synapse showed deficits in presynaptic short‐term plasticity, primarily short‐term facilitation (STF). To address this we applied trains of low‐ to high‐frequency extracellular stimuli to the str. lucidum (Fig. 6 *A*–*D*, left). Stimulation at all tested frequencies (5–100 Hz) resulted in no effect on the paired‐pulse ratio (PPR; first to second stimuli amplitudes) between control and *COX10*‐deficient mice (*P = *0.08, 2‐way ANOVA; Fig. 6 *E*). Likewise, there was no effect of *COX10* loss on STF (first to last stimuli amplitudes) at lower stimulation frequencies within the train of 10 stimuli (*P = *0.73, 0.30 and 0.19 for 5, 10 and 20 Hz respectively, 2‐way ANOVA, Fig. 5 *A* and *B*). However, 10 stimuli delivered at the highest frequencies (50 and 100 Hz) resulted in a substantial reduction in the EPSC amplitude following repetitive stimuli in complex IV‐deficient mice (Fig. 6 *C* and *D*), with the most marked reduction compared to control mice seen at 50 Hz. At frequencies above 20 Hz the EPSCs displayed a high STF in wild‐type mice (Fig. 6 *B*–*D*), as previously shown (Nicoll & Schmitz, 2005). Interestingly, in the complex IV‐deficient mouse, STF was significantly reduced at 50 and 100 Hz (Fig. 6 *E* and *F*). This reduction in STF was highly different between control and *COX10*‐deficient mice (*P < *0.0001 and *P = *0.005 for 50 and 100 Hz, respectively; 2‐way ANOVA). It should be noted that the most profound STF differences were present at low EPSC amplitudes (when EPSC < 100 pA; STF at 50 Hz = 7.6 ± 2.4 in control mice, 4 cells). Despite this, even taking into account this amplitude‐dependent effect, STF was much lower in *COX10*‐deficient mice (STF at 50 Hz = 3.0 ± 0.4, 8 cells, *P = *0.003). Furthermore, EPSC recordings performed in CA1 PCs, via stimulation of the CA3 Schaffer‐collateral synapses located within str. radiatum under identical recording conditions to that of the MFBs showed that deficits in presynaptic transmission do not transmit to the next synapse within the hippocampus (data not shown), indicating that the effect observed is specific to the presynaptic MF axons.

These data confirm that the MF–CA3 synapse is highly plastic, showing a high degree of STF, which is markedly reduced in the *COX10*‐deficient mouse as due to the high energy requirement of high‐frequency repetitive stimulation. The reduced STF at high frequencies and increased failure rates suggest a strong deficit in presynaptic, preventing correct synaptic transmission at the MF synapse.

### Loss of presynaptic complex IV results in reduced recruitment of local inhibition

**Figure 7:**
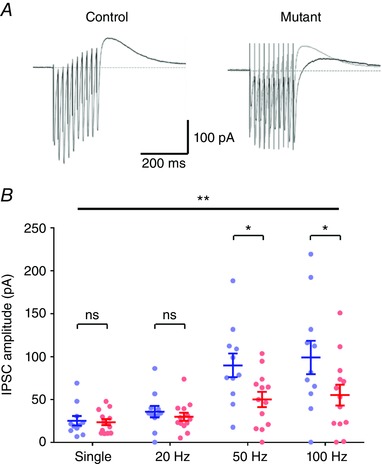
Loss of presynaptic *COX10* expression in DGCs leads to a strong reduction in local inhibition in CA3 *A*, representative compound IPSC evoked by 50 Hz stimulation in either control mice (left) or *COX10*‐deficient mice (right) recorded from CA3 PCs (outward current measured 50–100 ms following the last stimuli) showed that inhibition driven by stimulation of str. lucidum was much reduced, confirming that the DGC synapses onto local interneurons display similar deficits as the MFB. *B*, quantification of the effect of single stimulation and frequency trains on IPSC amplitude. Statistics shown: ns, *P > *0.05; ^**^ *P < *0.01; ^***^ *P < *0.001.

In many recordings we observed an outward current following both single stimuli and trains of stimuli to str. lucidum, representing a mixed IPSC composed of GABA_A_ and GABA_B_ receptor‐mediated currents. Given that DGCs form more than 10‐fold more synapses with GABAergic interneurons (INs) than with CA3 PCs (Acsády *et al*. 1998), this outward current may result from the recruitment of local GABAergic interneurons in CA3. To assess whether DG inputs to CA3 altered the inhibitory tone of CA3, we measured the mean IPSC amplitude following either single stimuli or trains of 20–100 Hz (Fig. 7). In control mice, the mean IPSC amplitude following single stimuli was 24.9 ± 5.5 pA, which was not significantly different from that of complex IV‐deficient mice (23.3 ± 3.5 pA, *P = *0.99, 2‐way ANOVA with multiple comparisons, Fig. 7 *B*). In agreement with the high‐frequency stimulation data for EPSCs, the degree of inhibition was reduced by ∼50% following 50 and 100 Hz trains in *COX10*‐deficient mice (*P = *0.03 and 0.01, respectively, Fig. 7 *B*), but not at 20 Hz (*P = *0.99).

**Figure 8:**
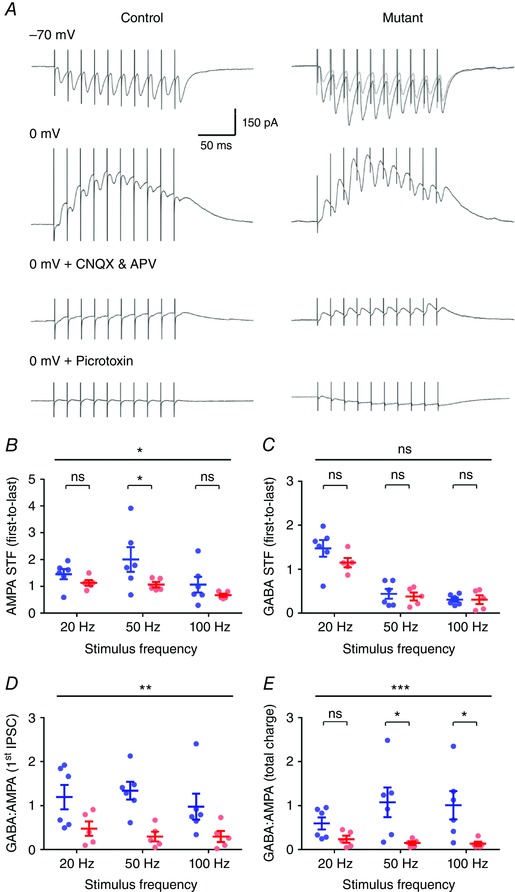
Loss of presynaptic *COX10* expression in DGCs leads to a dramatic loss of feedforward inhibition in CA3 PCs *A*, representative synaptic responses evoked by 50 Hz stimulation, in either control mice (left) or *COX10*‐deficient mice (right) recorded from CA3 PCs at the Cl^−^ reversal potential (–70 mV, top) or the AMPA/NMDA reversal potential (0 mV, middle). Recurrent inhibition was blocked with application of CNQX (10 μm) and dl‐APV (50 μm). The residual IPSCs measured at 0 mV were fully blocked by application of picrotoxin (50 μm). *B*, measurement of AMPA STF (first to last EPSC) at the Cl^−^ reversal potential in control (blue) and *COX10*‐deficient (red) mice (*n = *6 control cells and 5 mutant cells) at 20–100 Hz stimulus frequencies. *C*, quantification of the GABA STF profile, as recorded at the AMPA/NMDA reversal potential, according to the same scheme as *B*. *D*, measurement of the GABA:AMPA ratio of the first synaptic currents, over the same stimulus frequencies. *E*, GABA:AMPA ratios quantified for the total charge transferred, area under curve, for the same frequencies in both control and *COX10*‐deficient mice. Statistics shown: ns, *P > *0.05; ^*^ *P *< 0.05; ^**^ *P < *0.01; ^***^ *P < *0.001; from 2‐way ANOVA with multiple comparisons.

To confirm that these IPSCs were indeed mediated by feedforward inhibition, resulting from MF activation of local INs, and not direct stimulation of inhibitory axons, we performed voltage‐clamp experiments using a caesium gluconate‐based internal solution (Fig. 8). To measure accurately IPSC amplitudes we delivered a stimulus which produced an IPSC of approximately 150 pA (control: 162.1 ± 41.2 pA; *COX10*‐deficient: 145.1 ± 38.7 pA; *P = *0.88, Mann–Whitney test). EPSCs recorded in response to the same stimulus were substantially larger in *COX10*‐deficient mice (control: 118.0 ± 39.6 pA; *COX10*‐deficient: 414.2 ± 99.6 pA; *P = *0.02, Mann–Whitney test). Under these conditions, recording EPSCs at the Cl^−^ reversal potential (–70 mV) we observed a very similar pattern of STF to that for potassium gluconate experiments, whereby STF was low in *COX10*‐deficient mice (*P = *0.02, 2‐way ANOVA), most noticeably at 50 Hz, which had an STF ratio of 1.1 ± 0.1 (*n = *5 cells), compared to 2.0 ± 0.5 in control mice (*n = *6 cells; *P = *0.02, 2‐way ANOVA with multiple comparisons, Fig. 8 *B*). We next asked if the GABA:AMPA ratio of feedforward inhibition was reduced in the *COX10*‐deficient mice. To assess this we measured the first IPSC amplitude and total charge transfer at 0 mV, and subtracted residual monosynaptic IPSCs remaining in the presence of CNQX (10 μm) and APV (50 μm) from the pharmacologically naive IPSC at 0 mV. In both genotypes, there was a small residual current in the presence of CNQX and APV, due presumably to direct activation of GABAergic axon collaterals, which was not different between control and *COX10*‐deficient mice (*P = *0.18, Mann–Whitney test). The STF profile of feedforward IPSCs was not different between control and *COX10*‐deficient mice (*P = *0.20, 2‐way ANOVA, Fig. 8 *C*). The GABA:AMPA ratio of the first IPSC was reduced when comparing control and *COX10*‐deficient mice (*P = *0.0001, 2‐way ANOVA). To assess whether the total inhibitory drive was reduced in *COX10*‐deficient mice, we finally measured the total charge transferred over the time course of the stimulus train. *COX10*‐deficient mice showed a considerable reduction in total inhibition at both 50 and 100 Hz, with respect to total charge transfer (*P = *0.0005, 2‐way ANOVA); indeed the GABA:AMPA ratio was > 5 fold lower at both of these stimulation frequencies (*P = *0.02 and 0.03, respectively, Fig. 8 *E*).

These data show that in addition to reduced excitatory drive during high‐frequency stimulation, inhibition produced by the same stimulation of str. lucidum is even more strongly attenuated, suggesting that the deficit in presynaptic energy turnover extends to all synapses formed by DGCs, and will strongly perturb CA3 network function.

## Discussion

In the current study we show that mitochondrial respiratory chain complex IV deficiency, through conditional loss of protohaem IX farnesyltransferase achieved by selective removal of the *COX10* gene in DGCs, does not affect basal synaptic transmission of the DG–CA3 MFB synapse. However, local network excitability was increased and short‐term presynaptic facilitation markedly attenuated, in line with the cognitive decline seen in mitochondrial disorders in patients and rodent models. These data indicate that intact mitochondrial function, and thus ATP generation, is a prerequisite for correct synaptic function, and compromised short‐term plasticity and recruitment of inhibition associated with mitochondrial dysfunction may contribute to some of the cognitive phenotypes associated with neurodegenerative disorders.

### Loss of complex IV in DGCs dramatically impairs transmission at the DG–CA3

In the inducible Thy1‐cre mouse line utilized in this study, Cre‐recombinase shows regional differences in expression; notably DGCs, but not CA3 and CA1 pyramidal cells, of the hippocampus, leading to loss of complex IV in nearly all DGCs, leading to impaired mitochondrial function. It is clear from previous studies that reduced mitochondrial function has clear implications for the ability of DGCs to accumulate intracellular Ca^2+ ^(Lee *et al*. 2012) both at the soma and in presynaptic MFBs. Moreover, despite the loss of the *COX10* gene, the MFB synapse functions relatively normally in response to single or classic 20 Hz paired‐pulse stimuli, indicating that the function of DGCs is not fully compromised. However, as DGCs are known to fire bursts of action potentials at high frequencies, acting as sparse coding devices (Jung & McNaughton, 1993), the deficits in STF in complex IV‐deficient mice are highly pertinent. Furthermore, it is known that induction of CA3 PC firing *in vivo* requires high‐frequency DGC discharge > 20 Hz (Henze *et al*. 2002). Thus the effect of complex IV deficiency on sustained synaptic activity implies that the DGC axons are unable to maintain glutamate release to the same extent as control mice, due to reduced ATP turnover, leading to reduced CA3 PC firing.

### Increased activity of the local CA3 network in complex IV‐deficient mice

However, despite the clear deficit in MF transmission, CA3 pyramidal cells receive the vast majority of their synaptic contacts from other sources than DGCs, which on average form < 41 synaptic contacts onto a single CA3 PC (Henze *et al*. 2000), while commissural‐associative and perforant‐path synapses contribute the vast majority of synaptic inputs to CA3 PCs (Gould *et al*. 1990). We observed an increase in spontaneous EPSC amplitudes in CA3 PCs, suggesting potential synaptic remodelling at the level of the local microcircuit, perhaps due to the reduced synaptic drive onto CA3 PCs from DGCs. The local commissural‐associative pathway of CA3 is a likely source of compensation, through recurrent connections with greater release probability (Canepari & Cherubini, 1998). It is unlikely that additional synapses are formed, as we observed no change in spontaneous EPSC frequency. Nor did we observe any major change to intrinsic excitability of CA3 PCs that would lead to increased excitability. It is also possible that the increase in spontaneous activity was due to a circuitous interaction via CA1 and the entorhinal cortex. However, as the excitability of CA3 PCs was generally unaffected, and there was no major change in STF from CA3 to CA1, this seems unlikely.

The reduction in feedforward inhibition produced in CA3 PCs by stimulation of the MF pathway in str. lucidum will directly result in a reduced inhibitory profile of CA3 PCs, an effect which is critical for the maintenance of an inhibition/excitation balance, preventing epileptiform activity (Lawrence & McBain, 2003). Indeed, it has been shown previously that the majority of synapses formed by DGCs target INs at a ratio of 10:1 over CA3 PCs (Acsády *et al*. 1998), and therefore it is not surprising that feedforward inhibition was more strongly attenuated in the *COX10*‐deficient mice. In addition, the increased rate of putative unitary EPSC failures in the *COX10*‐deficient mice would suggest that local feedforward INs receive overall less excitatory input in the first instance, explaining why the first stimulus showed deficits in GABA:AMPA ratio. What the major postsynaptic targets of DGC inputs are is not fully clear and the source of feedforward synaptic inhibition was not identified, but it is known that the MF pathway converges onto many INs in CA3, including those within str. lucidum itself (Vida & Frotscher, 2000); the direct impact of complex IV loss on these interneurons would require further examination. Furthermore, loss of inhibitory drive may result in increased release probability from local CA3 recurrent synapses, through loss of presynaptic GABA_B_ receptor activation (Lüscher *et al*. 1997), leading to enhanced release probability at local CA3 synapses, which would require further examination. However, as it is known that reduced GABA:AMPA ratios are believed to be central to the development of epileptic syndromes (Fritschy, 2008), the strongly reduced inhibitory drive due to reduced feedforward inhibition may perhaps explain increased seizure prevalence in patients with neurodegenerative disorders (Poser & Brinar, 2003).

In summary, we have shown that presynaptic activity is substantially reduced in a mouse model of neurodegeneration that mimics human pathology and neuronal mitochondrial respiratory chain complex IV deficiency. The present findings suggest, despite maintenance of synaptic transmission, deficits are apparent in short‐term plasticity both in direct excitatory and indirect inhibitory neurotransmission, reducing network function, which may have deleterious consequences for cognitive function.

## Additional information

### Competing interests

The authors state no conflict of interest.

### Author contributions

All experiments were performed in laboratories located in the Centre for Integrative Physiology or the Centre for Neuroregeneration at the University of Edinburgh. Conception and design of the experiments: S.A.B., G.R.C., K.S.M., P.J.B., P.C.K., D.J.M. and D.J.A.W.; collection and assembly of data: S.A.B., G.R.C., K.S.M., D.J.M. and D.J.A.W.; analysis and interpretation of data: S.A.B., G.R.C., K.S.M., P.J.B., P.C.K., D.J.M. and D.J.A.W.; drafting the article or revising it critically for important intellectual content: S.A.B., G.R.C., P.J.B., P.C.K., D.J.M. and D.J.A.W. All authors approved the final version of the manuscript, and all persons designated as authors qualify for authorship and all those who qualify for authorship are listed.

### Funding

This work was funded by Medical Research Council (MRC) MR/K014137/1: S.A.B., P.C.K., D.J.A.W.; Biogen Idec 633MAH: G.R.C., D.J.H.; Progressive Multiple Sclerosis Challenge Award PA0051: D.J.M.; Medical Research Council (MRC) MR/L011379/1: P.J.B.; Wellcome Trust 107008/Z/15/Z: P.J.B.
